# Fuzzy Drug Targets: Disordered Proteins in the Drug-Discovery Realm

**DOI:** 10.1021/acsomega.2c07708

**Published:** 2023-03-08

**Authors:** Suman Saurabh, Karthik Nadendla, Shubh Sanket Purohit, Ponnurengam Malliappan Sivakumar, Sibel Cetinel

**Affiliations:** † Molecular Sciences Research Hub, Department of Chemistry, Imperial College London, London W12 0BZ, U.K.; ‡ Center for Misfolding Diseases, Yusuf Hamied Department of Chemistry, Lensfield Road, University of Cambridge, Cambridge CB2 1EW, U.K.; § Department of Clinical Haematology, Sahyadri Superspeciality Hospital, Pune, Maharashtra 411038, India; ∥ Institute of Research and Development, Duy Tan University, Da Nang 550000, Vietnam; ⊥ School of Medicine and Pharmacy, Duy Tan University, Da Nang 550000, Vietnam; ¶ Nanotechnology Research and Application Center (SUNUM), Sabanci University, Istanbul 34956, Turkey; # Faculty of Engineering and Natural Sciences, Molecular Biology, Genetics and Bioengineering Program, Sabanci University, Istanbul 34956, Turkey

## Abstract

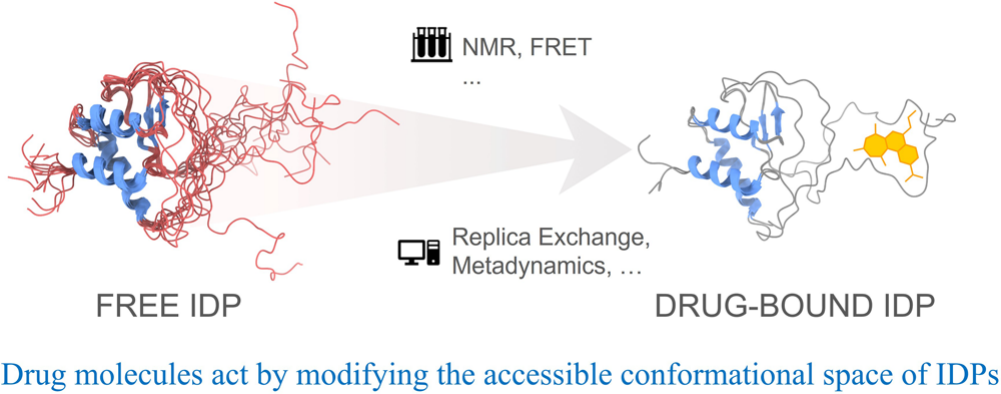

Intrinsically disordered proteins (IDPs) and regions (IDRs) form a large part of the eukaryotic proteome. Contrary to the structure–function paradigm, the disordered proteins perform a myriad of functions *in vivo*. Consequently, they are involved in various disease pathways and are plausible drug targets. Unlike folded proteins, that have a defined structure and well carved out drug-binding pockets that can guide lead molecule selection, the disordered proteins require alternative drug-development methodologies that are based on an acceptable picture of their conformational ensemble. In this review, we discuss various experimental and computational techniques that contribute toward understanding IDP “structure” and describe representative pursuances toward IDP-targeting drug development. We also discuss ideas on developing rational drug design protocols targeting IDPs.

## 1 Introduction

Intrinsically disordered proteins are the proteins that do not fold into a unique 3-dimensional structure.^1,2^ These proteins exist both independently (IDPs) and as domains (IDRs) in folded proteins.^1,2^ Disorder is more widespread in the eukaryotic proteome as compared to less complex life forms, suggesting that it is evolutionarily important.^3^ Around 60% of eukaryotic proteins are suspected to have long disordered domains. Even enzymes or proteins that rely on their unique 3-dimensional folded structure for their catalysis or function contain IDRs of sequence lengths similar to those in other folded proteins,^4^ which help them gain regulatory control over processes.

In general, the sequence of disordered proteins lacks order-promoting amino acids (Ile, Leu, Asn, Val, Phe, Cys, Tyr, Trp), most of which are hydrophobic, whereas they are rich in amino acids that are either polar or charged.^5^ The abundance of charged and polar amino acids, coupled with a lack of hydrophobic ones, results in disorder-promoting forces (interaction with surrounding water) that overwhelm ordering/compacting forces (resulting from hydrophobic interaction), leading to disorder.^5^ While IDPs are disordered, they feature intermittently folded regions. Both experimental and computational studies have shown that IDPs fold into transient structures.^6^ These transient structures might have a role to play in binding to their ordered partners.^6^

**Figure 1:**
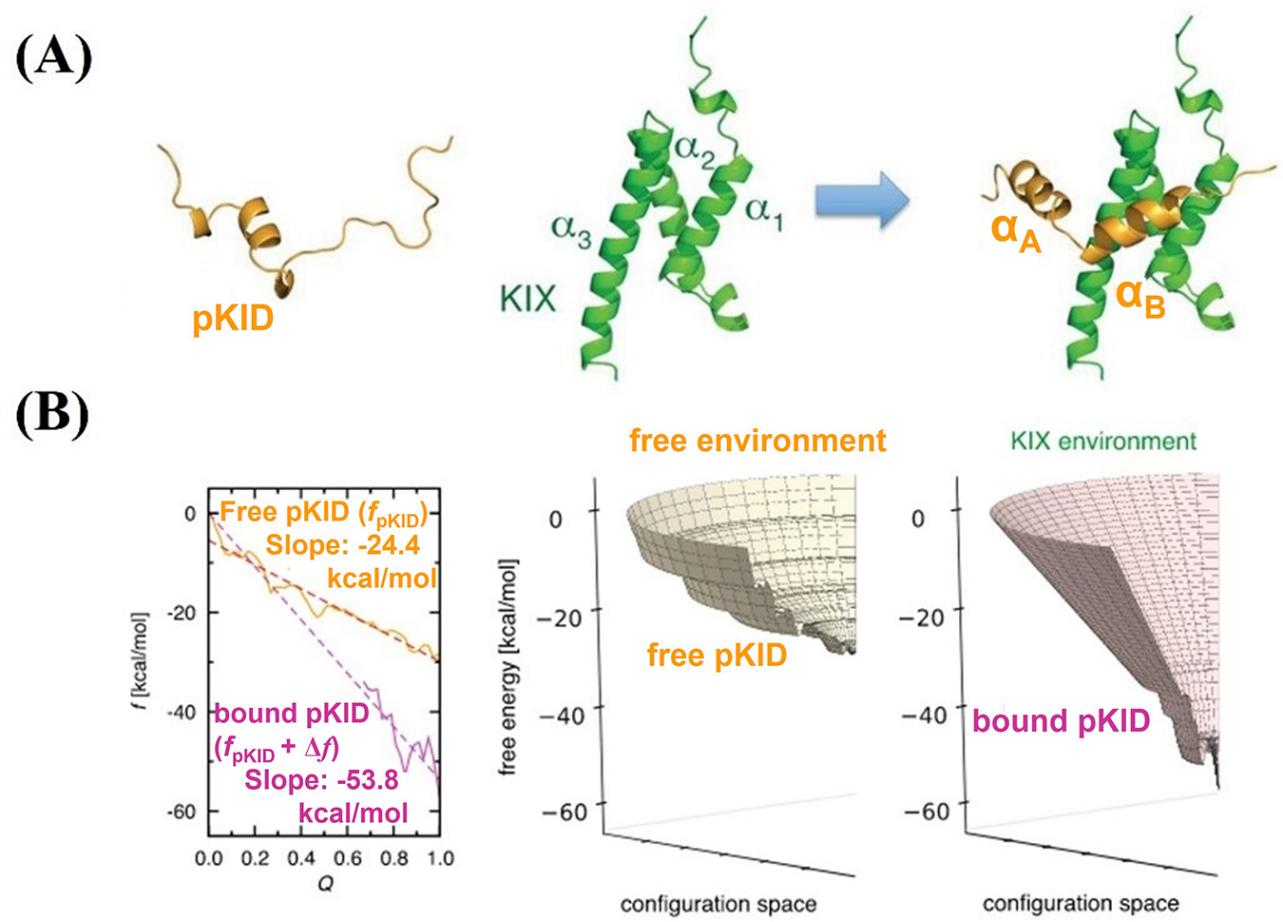
(A) Binding upon folding observed during pKID–KIX complexation.^14^ (B) The free energy profile as a function of the number of native contacts (*Q*) for the free (orange) and KIX-bound (magenta) pKID. The dashed lines are linear fits to the profiles. The free energy landscapes are also shown in a 3-D representation. Adapted with permission from *Sci. Rep.***2019**, *9*, 14927.

The conformational free energy surface of IDPs, unlike that of the folded proteins (which feature a deep minimum corresponding to a unique folded structure^7,8^), contains multiple shallow wells separated by weak energy barriers.^9,10^ The nature of the free energy profile renders the disordered proteins easily affected by environmental variables (like temperature, pH, salt concentration, and the presence of other proteins). The sensitivity of the structure to environmental factors results in phenomena like folding upon binding that have functional significance.^11,12^ The IDP–target interactions featuring coupled folding and binding result in highly specific but low affinity complexes, due to the inherent flexibility of IDPs and the consequent large entropy of binding.^13^ The free energy landscape of proteins that fold upon binding shows a continuous shift from a flat landscape with multiple shallow minima to a folded protein-like funnel-shaped landscape as the free protein undergoes transition to a complex-bound state. Unbiased molecular simulation of the pKID–KIX complex explicitly demonstrated the transition in the free energy landscape of pKID^9,14^ (see Figure 1) during the binding process.

Disordered proteins perform many important functions.^15−25^ The most important among them is their role in cellular signaling,^15^ the mechanism of regulation of cellular processes by a complex protein network through transmission of regulatory signals. The ability to attain markedly different conformations under different conditions allows the disordered proteins to play the role of molecular switches and hubs in signaling networks.^15,16^ Proper signal transmission demands a delicate balance between the specificity of protein–protein and protein–ligand interactions and their reversibility. Specificity is required to ensure a one-to-one relation between an initiating process and its outcome. Reversibility of the interactions allows the signals to be transient in nature. Interactions featuring coupled folding and binding (due to its large entropic cost) decouple binding specificity from the magnitude of binding free energy and result in highly specific but low affinity complexes that are reversible. In addition, the capability to bind to multiple targets, due to the structural plasticity, makes IDPs suitable for many-to-one and one-to-many signaling^15^ as constituents of complex regulatory networks. Protein–protein interactions involved in the regulation of cellular processes like transcription should result in high association rates and must have high dissociation constants as well in order to quickly initiate and switch off processes in response to signals. Regulation of transcription also requires the accessibility of sites for local chemical changes like post-translational modification (PTM). These requirements are satisfied by IDRs. The highly specific and low affinity complexes resulting from IDP–target interactions are associated with high dissociation rates. On the other hand, induced fit-based^26,27^ complexations involving IDPs and IDRs are characterized by high association rates. Due to the easy accessibility of amino acids in IDRs, like the histone tails, they can act as switches through a combination of PTMs and regulate transcription.^19−24^ Owing to structural plasticity and the nature of IDP–target interaction, around 70% of the signaling-related proteins^28^ in eukaryotes and half of the transcription factors^17,18^ are either IDPs or contain IDRs.

Thus, disordered proteins are clearly overrepresented in signaling networks. The sensitivity of conformations of these proteins to environmental conditions affects their ability to transduce signals and recognize partners. Mutations resulting from erroneous PTMs or abnormal alternative splicing^29^ would also affect their behavior. Cellular signaling relies on the specificity of IDP–target interaction. The specificity of the interactions would depend on IDP concentration. A large local concentration could overwhelm specificity and lead to nonspecific binding and toxicity. The protein degradation and clearance machinery keep a tight check on the concentration of disordered proteins, and a deregulation might lead to diseases. Environmental changes may also lead to the disordered proteins attaining conformations that act as precursors of aggregation, leading to amyloidogenic diseases. Owing to these factors, IDPs are overrepresented in many disease pathways and are also at the center stage of many diseases.^30−32^ For instance, the disordered protein p53 plays a central role in tumor suppression via cell apoptosis. A loss of function leads to cancer. Parkinson’s disease^33,34^ and certain other synucleopathies^35,36^ are caused by the accumulation of aggregates of the disordered protein α-synuclein in the neuronal cytoplasm. Aggregates of amyloid-β (Aβ) and tau peptides are involved in Alzheimer’s disease.^37,38^ Pathological aggregates of the prion protein result in encephalopathies like the Creutzfeldt–Jakob disease and scrapie.^39,40^ Huntingtin protein is implicated in Huntington’s disease^41,42^ and islet-amyloid peptide in type-2 diabetes.^43,44^ These are just a few examples, and the space of diseases involving disordered proteins is very large.^30,31^ Efforts to design drugs that target the protein–protein complexes at the root of disorder-related diseases are an active field of research. The approach to designing drugs, however, is different from that employed for targeting folded protein complexes. The lack of a well-defined 3D structure presents many issues, and a combination of experimental and computational tools must be employed to visualize the target.

In the following, we discuss in brief the experimental and computational techniques in regular use for the study of IDP conformation and their interaction with drug molecules, mention the ongoing efforts in force field development, and discuss the prominent efforts in discovery of drugs targeting disordered proteins and domains. We use the discussion to highlight the basic differences in the issues and approach to discover drugs that target interactions mediated by disordered proteins as opposed to those involving folded proteins. Finally, we discuss the possibility of rational drug design approaches with respect to IDPs.

## 2 Experimental Techniques in Use to Study IDPs

IDPs have high conformational entropy and therefore do not exist in a countable number of conformations. Instead, their structural behavior is studied by considering all possible conformations or an ensemble of conformations, which are usually represented as protein clouds.^45^ Therefore, a few conventional techniques may not be enough to structurally characterize IDPs. Instead, a combination of many techniques is generally employed to study their structure and interaction with drug molecules.

### 2.1 Techniques for Detecting Disorder and Determining the IDP Structural Ensemble

Various experimental techniques are in use to detect IDRs in proteins, determine the extent of disorder, and study the structural features of IDPs and IDRs. Mass spectroscopy (MS),^46,47^ for instance, involves generation of ionized proteins in a gaseous phase that can be profiled by tracking the protein trajectory in an applied electric or magnetic field. The output of the experiment is the relative protein population as a function of mass-to-charge ratio. Milder experimental conditions in soft ionization techniques like electrospray ionization (ESI)-MS and native MS make the detection of protein conformational changes, native intact multiprotein domains, and noncovalent interactions between proteins and ligands possible.^48−52^ The extent of ionization in proteins, during their transition from the liquid to gas phase, is highly dependent on their fold.^53^ Therefore, charge-state distributions of proteins can provide insights into their structure. Rigid proteins are observed to have unique charge-state distributions.^54^ On the contrary, a highly flexible protein like an IDP can potentially be protonated in a variety of ways, leading to the detection of several charge-state distributions.^55,56^ Ion mobility (IM) spectrometry is another tool within the mass spectrometry field which is gaining attention in the study of structures of IDPs. As each conformer of an IDP has the potential to ionize differently in ESI, application of a weak electric field can differentiate such conformational isomers in a neutral gas phase. Therefore, ESI is used in combination with IM and MS as ESI-IM-MS to study the structural ensembles of IDPs.^57^ Hydrogen–deuterium exchange is another reliable tool in the identification of disorder. Deuterium exchange occurs rapidly at amide and side chain functional groups in IDPs/IDRs which are free and completely exposed to the solvent,^58^ whereas hydrogens in structured protein regions (α-helices, β-sheets) or in the hydrophobic interior exchange at a slower rate,^59^ allowing the detection of disordered regions. Circular dichroism (CD) can also be used to detect disordered regions. It is one of the most common methods to determine protein secondary structure. CD measures differential absorption of left and right circularly polarized light (LCP and RCP) by the sample. Amino acids absorb light differently according to their (structural) environment. In general, absorption properties in the far-UV range (180–260 nm) are useful for secondary structure determination as it is absorbed by amide backbones. Since IDPs/IDRs do not have a distinctive secondary structure, the far-UV CD spectra of such peptides or proteins are less complex; i.e., the differential absorption of RCP and LCP lights is minimal.^60,61^ Accounts of application of CD to disordered proteins can be found in the literature.^62^

Small-angle X-ray scattering (SAXS) is a widely used method for characterizing ensembles of disordered conformations and monitoring time-resolved structural changes in solution.^63^ Rigid proteins can be crystallized, and their structure can be determined using X-ray crystallography. Disordered molecules like IDPs cannot form crystals; therefore, they are studied, while randomly oriented in solution, using SAXS.^64^ Diffraction patterns obtained with SAXS are not as sharp and characteristic as those of crystallographic diffraction; however, they contain a significant amount of structural information, albeit at low resolutions (∼20 Å). Conformational ensembles of IDPs can also be determined using techniques like Förster resonance energy transfer (FRET), which is the nonradiative transfer of energy from a donor fluorophore to an acceptor fluorophore in proximity. The energy transfer between the donor and acceptor results in the donor being less fluorescent in the presence of the acceptor. The FRET efficiency, i.e., the ratio of the number of excited donors transferring energy to the acceptor and to the number of photons absorbed by the donor, is dependent on the distance between the fluorophores. Thus, a measure of FRET efficiency can be used to study disordered conformations with the donor and acceptor fluorophores installed on the two termini of the IDP.^65,66^ As the rate of donor fluorescence decay increases in the presence of an acceptor, this reduced lifetime of the donor can be studied using fluorescence lifetime imaging microscopy (FLIM) to determine the fraction of interacting moieties or residual structural features of the IDP.^67^

NMR has been the most reliable method for determining the structure of small proteins (<40 kDa) in solution and conformational ensembles of disordered proteins.^68,69^ NMR-based structure determination involves the calculation of chemical shifts and the change in resonance frequency of NMR active nuclei (like ^1^H, ^13^C, and ^15^N) relative to standard values in an applied magnetic field. The chemical shifts depend on the local environment of the nuclei and provide information on the local structure and overall protein conformation when the protein is labeled with the nuclei. NMR is an efficient tool for studying various phenomena like internal mobility, protein–protein and protein–ligand interactions, and PTMs at site-specific resolution. It is an ideal method for characterizing the residual structure of IDPs and IDRs.^70^ Nuclear Overhauser effect (NOE)-based analyses rely on dipolar coupling which occurs through space rather than bonds and therefore are an efficient way to explore the conformation of disordered proteins.^71^ NOE provides information on short-range H–H interactions, residual dipolar coupling on the bond orientations, chemical shifts and *J*-coupling constants on bonds, dihedral angles, and geometry. This information can be combined with techniques like molecular mechanics to optimize and obtain a high-resolution conformational ensemble of an IDP, consistent with the above-mentioned experimental data.

In addition to the techniques discussed above, electrophoresis,^72^ size exclusion chromatography,^73^ dynamic light scattering (DLS),^74^ and ultracentrifugation techniques^75^ can also be used to detect and analyze disorder but are only partially suitable.^45,76−78^ The quality of information that a study demands typically guides the type of technique to be used. Simple methods such as electrophoresis and stability studies (acid or proteolysis resistance, etc.) can inform us about the presence of a disordered domain in the protein of interest. Furthermore, information such as the degree of compactness can be obtained using size exclusion chromatography or DLS or a combination of both chromatographic and light-scattering techniques. Spectroscopic methods generally provide more detailed information regarding protein structure. Techniques like CD spectroscopy, FRET, MS, SAXS, and NMR are used for studying interactions at atomic, residue, or domain scales. Although not easily accessible to all, the highest level of information is provided by techniques specialized for studying structural information such as atomic force microscopy^79−81^ and microED.^82^ The reader is referred to a review by Scott et al.^45^ for a broader discussion on the various IDP characterization techniques.

### 2.2 Methods to Study Drug–IDP Interaction

An important aspect of drug discovery is determining an initial set of molecules, through high-throughput screening (HTS) of ligand libraries, that have a favorable interaction with the target protein. In the case of an IDP, determining the binding site or binding mode of the drugs along the IDP sequence is also relevant for understanding the mechanism of action of drugs. FRET is a useful technique that can be used to perform HTS on large libraries of compounds simultaneously. Examples of NMR and surface plasmon resonance^83^ based HTS can be found in the literature.^84,85^ HTS of IDP-binding ligands is also possible with a yeast–two hybrid system, a tool developed to study protein–protein interaction by fusing the proteins of interest with DNA-binding proteins and coupling their interaction with initiation of transcription. Small molecules can affect the transcription by interfering with the binding of the proteins, implying favorable interaction of the ligand with the IDP.^86^ Drug-IDP/IDR interactions can also be studied using fluorescence polarization, a technique based on the excitation of drug–fluorophore conjugates with polarized light. Interaction with disordered macromolecules will retain the polarization of the emitted light. Fluorescence polarization also allows HTS and therefore is a useful technique in IDP drug discovery.^86,87^ Aggregation inhibitors may be screened using fluorescence-based assays for the determination of aggregation kinetics, which is an easy and inexpensive approach for HTS.^88^ While CD spectroscopy can help determine the drug-induced changes in IDP structure, its use is limited by its sensitivity. CD is also sensitive to the types of buffers and salts used, making its adoption difficult for HTS. Interaction between chiral proteins and achiral ligands leads to changes in absorption spectra, a phenomenon known as induced circular dichroism. Host–guest interactions and supramolecular events can be studied using this phenomenon.^89^ Other techniques such as surface plasmon resonance and isothermal titration calorimetry can be used for the determination of dissociation constants and thermodynamics of binding, respectively.^90,91^ Dissociation constants can also be determined from NMR experiments by studying the change in chemical shifts as a function of ligand concentration.^92^ SAXS can be used to study conformational changes induced by ligand–IDP interaction. Ultimately, molecular mechanisms of drug–IDP interactions can be studied using MS, NMR, and other structural biology techniques. HADDOCK^93^ (high ambiguity driven biomolecular docking) is an NMR-based approach that can be used to study the drug–IDP interaction. In this approach, a half-filtered NOESY is obtained from signals for ^1^H coupled with ^12^C on one dimension, and ^1^H coupled with ^13^C on the other dimension. Chemical shift perturbations are clearly seen when a ^13^C-labeled protein interacts with an unlabeled ligand. These experimental results are then used to obtain molecular models (using flexible docking) that correlate with the experimental data.^94^

## 3 Molecular Dynamics Simulations

Molecular dynamics (MD) simulations have played a great supporting role to experimental methods in determining the details of molecular level processes that occur in biological systems.^95,96^ With the advent of advanced computing platforms, increasing accuracy of the biomolecular force fields, and advanced simulation methods, the capability of MD simulation is increasing in this regard. Recently a billion-atom-large atomistic simulation of the chromatin fiber has been performed, allowing a glimpse of the DNA organization therein.^97^ MD simulations have been able to depict, in detail, the process of initially free drug molecules binding to pockets on a protein surface^98^ and determine the mechanism of action of drugs.^99,100^ The process of protein folding and the sequence of processes that lead to folding have been studied in detail using simulations.^101,102^ The role of simulations in understanding the behavior of IDPs is even more important as the experimental techniques usually encounter an ensemble of conformations, and it is hard to achieve a resolution good enough to distinguish between different conformational states. In such cases, a correct interpretation of experimental results requires significant molecular insight. The size of the ensemble and the accuracy with which the relative weights of different conformational states are reproduced in the simulations are of utmost importance when studying disordered proteins, their aggregation process, response to environmental factors, and interaction with drug molecules.

### 3.1 Enhanced Sampling Techniques

While MD simulations are an effective tool for atomistic visualization of processes, determination of molecular mechanisms, and subtle but functionally important structural changes, obtaining sufficient conformational sampling is an issue. Conventional MD simulations can only sample regions around a local energy minimum close to the initial conformation of the system. The energy barriers encountered in the free energy landscape of a disordered protein are not as high as some other systems that MD simulations deal with. Nevertheless, the estimated roughness of the disordered free energy landscape is greater than 5RT,^103,104^ which could lead to significant conformational trapping. Thus, an accurate visualization of conformations and a prediction of associated conformational weights, within a finite-time simulation, require significant acceleration of dynamics, necessitating the use of enhanced sampling methods. Notable among them are Replica exchange,^105,106^ Metadynamics,^107,108^ and Accelerated molecular dynamics.^109,110^

One of the most widely used enhanced sampling techniques in the context of disordered proteins is Replica exchange molecular dynamics (REMD).^105,106^ In this method, multiple parallel simulations are performed at different temperatures (replicas), and an exchange of conformations is attempted between neighboring replicas (based on the Metropolis scheme^111^) at fixed time intervals. A successful exchange attempt leads to the transfer of high temperature conformations to low temperature replicas, resulting in an enhancement of conformational sampling. Ganguly et al.^112^ used implicit solvent REMD simulations to study pKID–KIX interaction and demonstrated the role of pKID phosphorylation in coupled folding and binding. Efficient exchange of conformations between replicas requires an overlap between their potential energy distributions. The larger the size of the system, the larger the difference in energy for a given temperature difference, amounting to a smaller overlap between energy distributions. Thus, for a reasonably high exchange probability, the temperature difference between neighboring replicas needs to be small, resulting in the requirement of a large number of replicas to cover a given temperature range. This leads to an increase in computational cost of the simulation, especially for explicit solvent systems. For IDPs, observing transient secondary structure elements requires a proper treatment of protein–water interaction, necessitating an explicit treatment of the solvent. To address the issue of computational cost, different flavors of REMD have been devised. Among them, the notable one is Hamiltonian Replica exchange (HRE),^113^ in which scaled Hamiltonians are used to obtain reasonable exchange probabilities. In REST,^114−116^ a special form of HRE, the Hamiltonian for the *m*^th^ replica (*E*_*m*_) has the form: , which features scaled solute–water (*E*_SW_) and water–water (*E*_WW_) interactions, and the subscript 0 corresponds to the lowest replica, for which the Hamiltonian, *E*_0_, is equal to the original Hamiltonian of the system. This form of the Hamiltonian leads to a considerable increase in exchange probability, even for large differences in replica temperatures, resulting in the requirement of a much lower number of replicas to cover a given temperature range as compared to original REMD. The method has been used to generate large ensembles of IDPs.^117^ Recent REST simulations^118^ performed in explicit water for three different IDPs (Histatin, Sic 1, and SH4UD) have shown that REST reproduces IDP ensembles consistent with NMR, SAXS, and SANS measurements. The ensembles were also found to properly represent the transient helices in the IDPs.^118^ A variant of this method is called Replica exchange with solute scaling (REST2),^119^ in which a slightly different Hamiltonian scaling scheme is used. The REST2 scaling scheme is known to increase the exchange efficiency over REST and thus leads to a requirement of an even lower number of replicas. Ramis et al. applied REST2 to a coarse-grained model of α-synuclein.^120^ Peng et al. used REST2 on the disordered protein human amylin and studied its conformational ensemble in comparison with experiments.^121^ Efforts to modify REST in order to further improve its performance are ongoing, resulting in more efficient methods.^122^

Metadynamics^107,108^ is another enhanced sampling technique in which a system is time evolved, and at appropriate intervals, a Gaussian potential hill is added, centered at the current location of the system along the collective variable of interest, forcing the system to visit new locations. With repeated addition of such hills, the local energy well gets filled up with Gaussian hills, and the system diffuses and falls into another nearby energy minimum where the process of addition of the Gaussian hills continues. The system can thus cross energy barriers and explore larger regions of the free energy surface. Han et al. used metadynamics to simulate coupled folding and binding of the disordered protein α-MoRE with its binding partner XD. Implementation of metadynamics^123^ and variants of this method^124^ to disordered proteins has been found to result in significant enhancement of conformational sampling. NMR-guided metadynamics has been used to characterize the free energy landscape of disordered proteins.^125^ Metadynamics has also been used to estimate the effect of small-molecule binding on the free energy landscape of cMyc.^126^

Accelerated MD (aMD)^109,110^ is an enhanced sampling method that relies on the modification of the free energy surface in a way that the depths of free energy wells are reduced, allowing for an easier surmounting of energy barriers. The conformational sampling obtained on the modified free energy surface is reweighed to trace back the sampling on the true free energy surface. The discovery of raltegravir, which is the first clinically approved inhibitor of HIV integrase, is attributed to this approach.^127^ aMD simulations have shown that the method improves chemical shift predictions for disordered domains, in agreement with NMR experiments.^128^

The IDP ensembles generated using these enhanced sampling methods, and MD simulations in general, can be used to perform docking to screen small-molecule ligands in a rational approach to drug discovery. An example of this approach will be discussed later in section 4.6. In addition to those discussed above, methods like umbrella sampling^129,130^ can be used to calculate accurate IDP–drug and IDP-folded protein binding energies and conformational free energy landscapes. Ithuralde et al.,^131^ for instance, performed umbrella sampling simulations to study the coupled folding and binding of c-Myb with CBP and the associated free energy surface. Such calculations in the presence and absence of drug molecules can help quantify the relative effect of different drug molecules on IDP–partner binding. The various enhanced sampling techniques in use for studying the structural ensemble of disordered proteins have been reviewed by Schor et al.^132^

### 3.2 Molecular Dynamics Force Fields for Disordered Proteins

Molecular dynamics force fields (*ff*s) were traditionally developed to study folded proteins. Thus, these *ff*s usually tend to present an overcollapsed picture of IDPs.^133,134^ In addition, the propensities of different secondary structure elements obtained from simulations employing these *ff*s are found to be higher than expected.^133^ Protein *ff*s thus need to be modified to be able to produce IDP conformational ensembles in agreement with experiments. The issue of a higher secondary structure propensity has been addressed by modifying the force field potential term corresponding to the backbone dihedral. The strategies involve training the dihedral term against coil dihedral data. This strategy leads to a proper dihedral sampling of coil-forming stretches of amino acids (*aa*s). This strategy has been utilized in the modified CHARMM *ff* CHARMM22*,^135^ AMBER *ff*s ff03*,^136^ and ff99SB*^136^ in combination with the TIP3P^137^ water model (*wm*), ff03ws^138^ in combination with the TIP4P/2005^139^*wm*, OPLS *ff*s OPLS-AA/M,^140^ and OPLS3.^141^ CHARMM22* was found to reproduce a characteristic β-hairpin structure in β-amyloids.^142^ CHARMM22*, in another study, reproduced the conformational ensemble of human amylin in close agreement with NMR experiments.^121^ Another method employed to improve the secondary structure representation for IDPs is the inclusion of the grid-based CMAP^143,144^ corrections to the dihedral energies, aimed at improving the agreement of the potential energy landscape in the ϕ−ψ conformational plane to quantum mechanically derived landscapes, which the usual sinusoidal dihedral potential function is unable to reproduce. CHARMM27 *ff*,^143^ which includes the CMAP correction to the CHARMM22 parameters, reproduces the correct helix–coil balance but does not reproduce hairpin structures. Kundu et al.^145^ found that the *ff* overstabilized the α-helix in α-synuclein. An improved version of the *ff*, CHARMM36,^146,147^ contains improved CMAP corrections derived from experimental NMR data. A variant of this *ff*, CHARMM36m,^148^ uses specific CMAP parameters for Gly and Pro. ff99SB-ildn^149^ has been modified to generate the a99sb-disp^150^ parameters, which provide a good description of both folded and disordered proteins with the TIP4P-D^151^*wm*. ff03CMAP^152^ is an example of another *ff* that works for both folded (in combination with TIP4P-Ew^153,154^) and disordered proteins (in combination with TIP4P-D). Some other *ff*s like ff14IDPSFF^155,156^ (modified from AMBER ff14SB) and CHARMM36IDPSFF^157,158^ (modified from CHARMM36m) are CMAP-based and IDP-specific. The latter has been shown to reproduce the experimental conformational ensemble of multiple disordered proteins.^159^ In a recent study, Privat et al. found that ff14IDPSFF could accurately predict experimental chemical shifts for α-synuclein.^160^ While ff14SB preserved the α-helices in α-synuclein, ff14IDPSFF predicted increased disorder and indicated the formation of β-sheet structures.^160^ Other strategies involve adding CMAP corrections to both side chain and backbone dihedrals (RSFF2C^161^) and adding corrections based on the chemical environment around a given *aa* (ESFF1^162^). Rahman et al.^142^ found that the IDP-specific force fields obtain more reasonable results for pure IDPs.

The issue of overcollapse has been addressed by modifying the water–protein interaction, keeping the protein–protein and water–water interactions unchanged. Early efforts in this area include the development of a dispersion-enhanced water model, TIP4P-D, that was able to generate proper conformational weights for the collapsed and extended states in combination with the AMBER and CHARMM *ff*s. Piana et al.^151^ found that for different disordered proteins, *ff*s OPLS, CHARMM22*, AMBER ff03, and ff99SB-ildn,^149^ in combination with the TIP3P water model lead to compact structures with radii of gyration in complete disagreement with FRET data. However, CHARMM22* was found to be close to experiments for strongly charged proteins.^151^ The TIP4P-D water model, which has a dispersion term at least 50% higher than those of the commonly used water models, led to conformations that match well with experiments for the AMBER and CHARMM *ff*s.^151^ Comparison of SAXS data with MD simulations of proteins containing IDRs has shown that TIP3P and TIPs3P water models lead to unusually compact structures.^163^ The radius of gyration of MAP2c measured from MD simulations with the TIP3P water model in combination with AMBER ff99SB-ildn was found to be 1.5 nm, much lower than the SAXS measured value of 2.5 nm.^163^ On the other hand, with the TIP4P-D water model, in combination with multiple *ff*s, the protein size was comparable to the experiments. With another IDR-containing protein, RD-hTh, the simulations performed using TIP4P-D agreed with experiments only when used in combination with the CHARMM36m^148^*ff*. It was found from microsecond long simulations that CHARMM36m in combination with TIP4P-D was most efficient in preventing the collapse of proteins not containing a large proportion of similarly charged *aa*s, whereas the AMBER ff99SB-ildn in combination with the TIP4P-D water model predicted the long-distance contacts most accurately.^163^ CHARMM36m with TIP4P-D was also seen to retain the transient α-helical structures better than other *ff*s in combination with TIP4P-D.^163^ Rieloff and Skepo^164^ performed a comparison of *ff–wm* combinations for the phosphorylated IDPs. These systems are relevant for studying the aggregation process of phosphorylated pathological IDPs like the tau protein, which aggregates in its phosphorylated form.^165,166^ They found that the CHARMM36m-TIPs3P combination led to overly compact structures, while the ff99SB-ildn/TIP4P-D combination matched the SAXS data better. In addition to the *ff–wm* combination, an aspect that needs to be considered in the case of IDPs is the protein charge and its dependence on IDP conformation. In the case of folded proteins, the protonation states of the titrable *aa*s are usually fixed at the beginning of the simulation using methodologies like PropKa.^167−169^ Such a prescription might not work for the IDPs. The p*K*_a_ of the titrable amino acids depends strongly on their molecular environment, which for an IDP will fluctuate rather frequently in correlation with conformational changes. Thus, methodologies like constant pH MD^170,171^ are relevant to such systems. Developments in MD methods for IDPs and force fields have been reviewed recently.^172^

## 4 IDPs Involved in Disease Pathways and Efforts in Drug Discovery

IDPs are postulated to be either directly implicated in many diseases or as parts of disease propagation pathways. The diseases range from cancer to neurodegenerative diseases.^173^ The most (in)famous example is Alzheimer’s disease,^37^ a form of dementia, caused by the aggregation of amyloid-β (Aβ) peptides in the neuronal regions. The Aβ peptides are of different types based on their sequence length which varies between 39–43 *aa*s. Among these, Aβ42 is the most amyloidogenic and has an intrinsic tendency to aggregate, leading to toxic fibrils. Another example is Parkinson’s disease^174^ that is caused by aggregates of α-synuclein^33^ blocking the dopamine-producing neurons. Transcription of larger than 36 repeats of the CAG trinucleotide sequence leads to the production of pathological Huntingtin’s proteins^175^ which undergo aggregation.^176^ The aggregates block basal ganglia, resulting in movement disorder characteristic of Huntington’s disease.^177^ Aggregates of the islet-amyloid peptide^178,179^ in the pancreas are thought to lead to unfolding of insulin resulting in type-2 diabetes.^43^ In addition to these aggregation-related disorders, many other diseases involve IDPs through various kinds of protein–protein interfaces (PPIs). The drug discovery efforts for IDP-related diseases involve targeting these PPIs formed by the interaction between IDPs and their binding partners. Lack of well-defined binding pockets, involvement of PPIs with a large area, and multiplicity of interactions lead to the need for multiple situation-specific approaches for designing drugs that target disordered proteins. Four different scenarios are usually encountered based on the nature of the PPI involved:

- Some disordered proteins undergo a disorder-to-order transition upon binding to their partners (coupled folding and binding). Such complexes can be formed between a disordered and a folded protein or between two disordered proteins.
- When directly targeting the active site is not possible, drugs can be designed to bind the regulatory disordered regions of otherwise folded enzymes involved in disease pathways, to suppress the enzyme activity allosterically.
- The third scenario is that of disordered or fuzzy complexes of two IDPs not featuring any folding upon binding.
- IDPs can form disease-causing aggregates and can be targeted by small-molecule drugs that bind to monomeric IDP and preclude aggregation.

In the following sections we discuss experimental and computational studies related to the discovery of drugs targeting IDPs and IDRs that are a part of the above-mentioned PPIs and through them demonstrate the application of various experimental and computational techniques discussed in earlier sections, in IDP-related drug discovery.

### 4.1 Targeting Protein–Protein Complexes Featuring Folding upon Binding

#### The cMyc**–**Max Complex

Many proteins need to bind to other proteins to attain an active state and perform their function. Such proteins might contain IDRs that act as the mediators in binding. An aberration in such interactions, for instance, the overexpression of one of the binding partners, might shift the complexation–decomplexation equilibrium and lead to an overdrive in activity, resulting in diseases. In such cases, the underlying binding needs to be hindered. A unique property of the interaction of an IDP/IDR with its interaction partners is the occurrence of folding upon binding. One of the strategies used to design drugs that hamper IDP–partner binding in such cases is to introduce molecules that can stabilize the disordered state and consequently prevent binding by making the accompanying folding free-energetically unfavorable. Drugs targeting cMyc–Max binding are one of the best examples of this approach. cMyc is a transcription factor, overexpressed in a variety of cancers, and thus is a drug target of interest. For performing its function(s) it needs to bind to the protein Max. The binding takes place through two basic helix–loop–helix leucine zipper (bHLHZip) domains on both proteins. The domains are intrinsically disordered in their monomeric form and undergo folding upon binding. Yin et al.,^180^ using a high-throughput assay, identified 7 structurally diverse small-molecule drugs that could bind to cMyc bHLHZip with high specificity. Hammoudeh et al.,^86^ using circular dichroism (CD), fluorescence polarization, and NMR, found that these small-molecule drugs demonstrate specific binding to three distinct sites along the bHLHZip domain of cMyc. CD spectra of the free and drug-bound states showed that different inhibitors affect the structure of the region to which they bind by different degrees. The study indicates that most of the inhibitors bind to a region (sequence: YILSVQAE) that is disordered in monomeric cMyc but lies at the PPI of the Max–Myc complex, suggesting that the molecules block the interaction between the two proteins. The fact that a single short stretch of amino acids can bind to a diverse set of molecules suggests that a rational approach to identifying small-molecule drugs that target disordered proteins is possible based on their sequence characteristics. The rational approach would involve generating a database of small stretches of sequences, from a large set of disordered proteins, that act as binding sites for a diverse variety of small molecules. The database can be used to determine general characteristics of such sequences. This would help predict prominent small-molecule binding sites on any IDP of interest. Small molecules that bind to these regions can then be found by screening through libraries and modified for improved binding efficiency and specificity.

MD simulations provide further insight into the binding between cMyc and the inhibitor molecules. While the experimental results discussed above suggest that the binding between cMyc and its inhibitors takes place at specific regions along the protein sequence, Jin et al.^181^ used MD simulations to show that the interaction between cMyc and one of the 7 inhibitors discovered by Yin et al. (named: 10074-A4) follows the “ligand cloud around protein cloud” picture, wherein the drug molecule interacts with cMyc at multiple regions along its sequence. The interaction of the ligand was transient, dynamic, and nonspecific. The snapshots obtained from simulations depicted the drug molecule interacting with multiple conformations of cMyc through three distinct regions along the cMyc sequence. One of the regions matches with the binding site determined by Hammoudeh et al.^86^ It was seen that cMyc attained more compact conformations in the presence of the inhibitor. It is probable that the inhibitor acts by restricting the conformational space of the IDP to a subset of the free IDP conformational space that is structurally incapable of interacting with Max.

#### Hindering p27-Cdk2/Cyclin A Binding

p27 is a disordered protein that regulates the function of cyclin dependent kinase (Cdk).^182^ Kinases are enzymes that facilitate the transfer of phosphoryl groups from donors like ATPs to acceptor substrates, leading to the phosphorylation of the substrates.^183^ Cyclin-dependent kinases (Cdk’s) are kinases that depend on the binding to the protein cyclin for their activity. Cdk plays a regulatory role in eukaryotic cell division.^184,185^ The N-terminal kinase inhibitory domain of p27 (p27^*KID*^) binds to the Cdk2/cyclin A complex and regulates its activity. An inappropriately phosphorylated form of p27 is implicated in breast cancer. Iconaru et al.^85^ studied small-molecule inhibition of the interaction of p27^*KID*^ with the CDk2/cyclin A complex.

**Figure 2:**
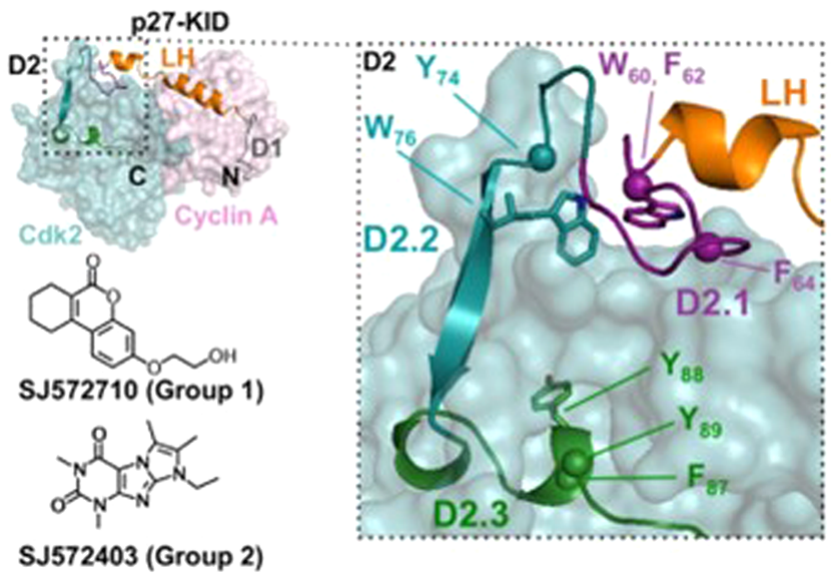
Structure of p27^*KID*^ bound to Cdk2/cyclin A (PDB ID 1JSU); the subdomains of p27^*KID*^, including D1, LH, D2.1, D2.2, and D2.3, are indicated. Chemical structures of two small molecules, SJ572710 (SJ710) and SJ572403 (SJ403), that bound to p27^*KID*^ and are members of group1 and group2, respectively, are shown. Adapted with permission from *Sci. Rep.***2015**, *5*, 15686.

p27^*KID*^ is disordered in the free state and undergoes folding upon binding to Cdk2/cyclin A (see Figure 2). The bound conformation of p27^*KID*^ can be divided into three distinct domains, D1, D2, and LH (see Figure 2). The D1 and D2 domains bind to cyclin A and Cdk2, respectively, and are connected by the LH domain. NMR-based screening of small molecules led to the identification of 36 molecules that bound to p27^*KID*^. During NMR screening the largest chemical shift perturbations were observed for residues in domain D2, whereas no perturbation was observed for the D1 and LH domains. The small molecules were divided into two groups: one group (group1) contained molecules that bound to subdomain D2.3, and the other group (group2) contained molecules that bound to all three subdomains, D2.1, D2.2, and D2.3 of D2 (see Figure 2). The amino acid composition of the subdomains is shown in Figure 2. Pharmacophoric modeling of the small molecules in these groups was used to identify additional molecules that could potentially bind to p27^*KID*^. One of the molecules from group2, SJ403 (see Figure 2), was shown to affect p27^*KID*^-Cdk2/cyclin A binding and increase kinase activity. One notes that the regions that interact with the small molecules are rich in aromatic amino acids. In addition it was found that the chemical shift perturbation on tryptophan (W) residues was higher than neighboring tyrosines (Y) and phenylalanines (F), suggesting a preferential interaction of the molecules with the indole ring of tryptophan. Mutation studies and MD simulations were performed to determine the molecular basis of the recognition of group1 and 2 molecules by p27^*KID*^. It was found that when W60 (of D2.1) and W76 (of D2.2) are mutated together to F or alanine (A) the chemical shift perturbations in the D2.3 subdomain, due to group2 molecules, are suppressed. A single mutation of either W60 or W76, however, did not lead to this effect. This observation suggests a higher importance of W60 and W76 and cooperativity between the subdomains in small-molecule binding. Mutation of Y88 (of D2.3) to A leads to no drug-binding-driven perturbation in subdomain D2.3, suggesting a higher importance of Y88 in drug binding. MD simulations revealed compact and extended conformations of p27^*KID*^. The compact conformations were characterized by clustering of Y88 (D2.3) and W60 (D2.1) or W76 (D2.2). It was inferred, based on the results of the mutation studies discussed above, that group2 molecules bind to compact conformations simultaneously engaging subdomains D2.1 or D2.2 and D2.3, while group1 molecules bind subdomain D2.3 on extended p27^*KID*^ conformations that do not feature subdomain clustering. Thus, group2 molecules can selectively stabilize compact conformations of p27^*KID*^ that are incapable of binding to Cdk2/Cyclin A.

**Figure 3:**
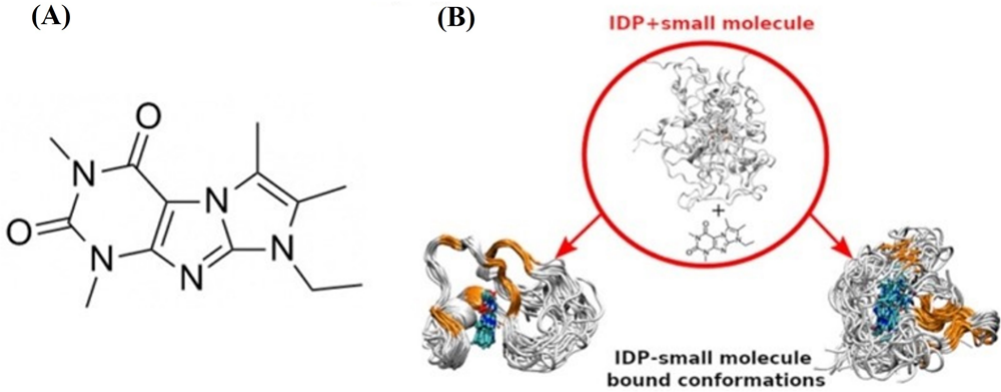
(A) Structure of SJ403. (B) The interaction of SJ403 with p27 restricts the conformational sampling of p27. The figure shows some binding conformations of p27 with the small molecule. The two ensembles shown correspond to two different metastable states of the drug–IDP complex and differ in the number of contacts between the IDP and SJ403. A conformational restriction of the protein in the presence of SJ403 is evident. The binding-induced rigid regions are shown in orange. SJ403 is shown in blue. Reprinted (adapted) with permission from *J. Chem. Inf. Model.***2020**, *60* (10), 5003–5010. Copyright 2020 American Chemical Society.

The system has also been studied using MD simulations. Herrera-Nieto et al.^186^ performed MD simulation of the interaction between the disordered D2 domain of proteins p27 and SJ403^186^ (see Figure 3). They used CHARMM22* *ff* with the TIP3P *wm* to perform multiple ∼100 ns long simulations for the protein and protein–ligand systems. They further use adaptive sampling algorithms to select conformations of interest from these simulations as starting conformations for new simulations. The protein–ligand and intraprotein contact information was used as the deciding parameter to select conformations. The collected data amounted to 6688 simulations of the p27–SJ403 complex and a cumulative time of around 535 μs, whereas the protein-alone data set had 8900 simulations and a cumulative time of 712 μs. Comparing the conformational space explored by the free and the ligand-bound protein, they infer that the small molecule restricts the conformational space explored by the protein (see Figure 3), while the conformations explored in the presence of SJ403 were also explored in its absence. Thus, the small molecule restricts the conformational space to a subset of the conformations that the free protein attains. In addition, it was seen that the region of the conformational space explored in the presence of SJ403 was completely disjoint from the region corresponding to partially folded conformations. Thus, the addition of the small molecule prevents specific intraprotein contacts that would be present in the partially ordered target-bound p27. It was found that the most populated p27–SJ403 bound state featured interaction between the drug and two TRP residues (W60 and W76) of p27. These are the same amino acids that showed the largest chemical shifts in NMR experiments described above^85^ (see Figure 2). This work presents a classic example of how MD simulations aid in a deeper understanding of the underlying mechanisms in the IDP–drug binding process.

#### N-Terminal Domain of Androgen Receptor

**Figure 4:**
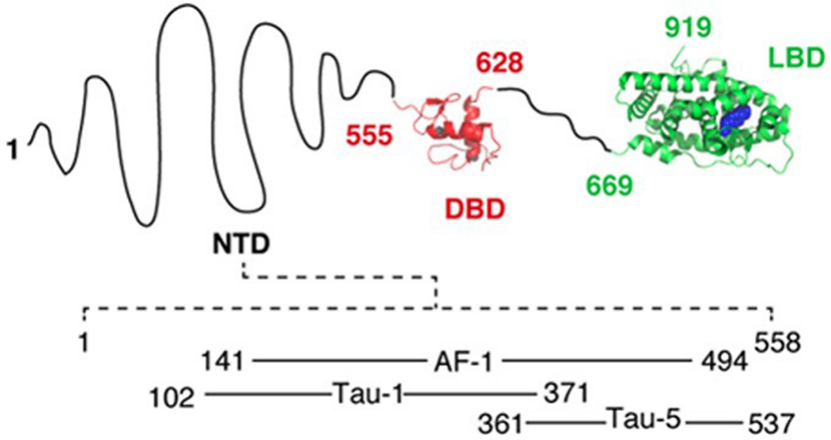
Predicted properties of the sequence of the transactivation domain of AR. The positions of the activation function-1 (AF-1) and transcription activation units 1 and 5 (Tau-1 and Tau-5) are depicted within the disordered N-terminal domain (NTD). The DNA-binding domain (DBD) and the ligand-binding domain (LBD) of AR are shown. Reprinted (adapted) with permission from *ACS Chem. Biol.***2016**, *11* (9), 2499–2505. Copyright 2016 American Chemical Society.

De Mol et al.^187^ studied the mechanism of action of EPI-001,^188^ a small molecule that is effective against castration-resistant prostate cancer (CRPC). CRPC is a condition that develops in patients undergoing androgen deprivation therapy as a treatment for prostate cancer. In this condition, cells begin to utilize alternative mechanisms for the activation of the androgen receptor. The interaction of the N-terminal domain (NTD) of the androgen receptor with various transcription factors is known to be a target in controlling CRPC. Rational drug design for this region is precluded by a lack of insight into the structure of the NTD. The domain is rich in order-inhibiting amino acids and contains alternating regions of low sequence complexity (polyGly, polyPro, etc.). However, some intermittent regions, with a low disorder propensity, rich in hydrophobic *aa*s are also present, which are known to be important for interaction with binding partners. In fact, certain regions (possibly the ones with low disorder propensity) of the NTD (in the AF-1 domain, see Figure 4) are known to undergo folding upon binding to their partners.^189^ It was found, using solution NMR, that EPI-001 interacts specifically with a partially disordered region of the transactivation domain of the androgen receptor, the transactivation unit 5 (Tau-5) (see Figure 4). The Tau-5 region of the NTD plays an important role in prostate cell proliferation in the absence of androgens (the activators of the androgen receptor) during androgen deprivation therapy.

**Figure 5:**
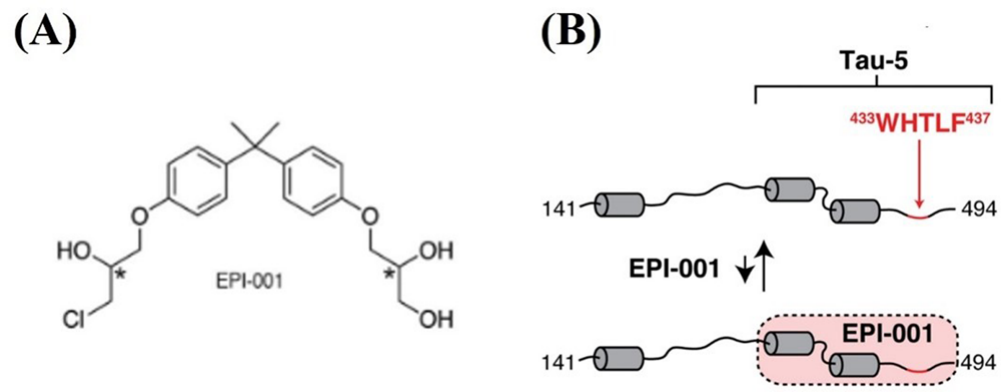
(A) Chemical structure of EPI-001. (B) Schematic showing the Tau-5 region of an androgen receptor and the regions where EPI-001 binds. Two of the three cylindrical features represent the partially folded helical domains of the Tau-5 region. The WHTLF region shown in red lies in the third region of the Tau-5 domain predicted to have high helical propensity and is known to be functionally important. Reprinted (adapted) with permission from *ACS Chem. Biol.***2016**, *11* (9), 2499–2505. Copyright 2016 American Chemical Society.

NMR measurements on AF-1 (see Figure 4) indicated three partially folded helical regions in the Tau-5 region separated by proline and glycine-rich disordered regions (see Figure 5).

The presence of EPI-001 (see Figure 5) causes small ^15^N chemical shifts in many Tau-5 residues in these three partially folded helical regions. Other partially helical regions of AF-1 (in the Tau-1 region, see Figure 4) showed negligible changes in resonance. The observation that a large number of residues in partially folded regions of Tau-5 showed NMR shifts and that the residues in similar regions of Tau-1 were not affected suggests the presence of a subtle molecular recognition mechanism in the binding of EPI-001 to Tau-5, where the small molecule would selectively interact with an ensemble of Tau-5 conformations and not with specific regions along the sequence. The experiments, however, cannot ascertain whether the interaction with EPI-001 broadens the conformational ensemble of Tau-5 by driving the domain to attain entirely new conformations, or it selects a subset of suitable conformations out of the range of conformations that Tau-5 attains. The determination of the molecular recognition mechanism in such detail would require techniques like MD simulations in combination with enhanced sampling techniques, similar to what has been discussed earlier in the example of protein p27.^186^

### 4.2 Allosteric Inhibition of Enzymes by Targeting Disordered Domains: Inhibition of Protein Tyrosine Phosphatase 1B

**Figure 6:**
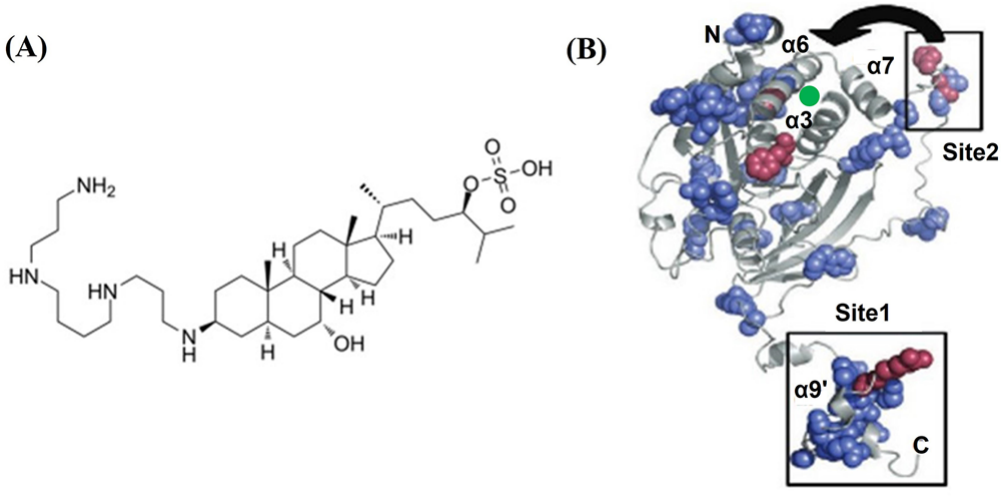
(A) Chemical structure of MSI-1436. (B) A single conformer of PTP1B_1–393_ with residues colored based on the chemical shift perturbations. The blue residues fall within one standard deviation from the mean chemical shift perturbation (mean + σ), while the red ones fall within three standard deviations from the mean. The two MSI-1436 binding sites are shown. The proximity of the second binding site to an allosteric site is shown by an arrow. A low affinity MSI-1436 binding site is marked with a green circle. Adapted with permission from *Nat. Chem. Biol.***2014**, *10* (7), 558–66.

Protein tyrosine phosphatase 1B (PTP1B) is overexpressed in breast tumors. Experiments show that it plays an active role in promoting signaling events that lead to breast tumorigenesis.^190^ In addition, it is established as a viable target in diabetes and obesity.^191^ The prospect of inhibiting PTP1B by targeting its active site is precluded, as potential inhibitors that block the active site were found to be strongly charged.^192^ Alternative ways of controlling PTP1B activity thus needed to be explored. Krishnan et al.^193^ found that the small-molecule MSI-1436, also known as Trodusquemine (see Figure 6), could inhibit PTP1B activity. They used FRET and NMR to determine the binding site of the small-molecule inhibitor on PTP1B and determined its mechanism of action. It was found that, while MSI-1436 inhibited both the catalytic domain (residues 1–321) and the full PTP1B (residues 1–405), the small molecule had a 7-fold higher affinity for the full PTP1B, implying that the prominent binding site of the molecule lies in its C-terminal domain. Dynamic light-scattering measurements showed that the radius of hydration of PTP1B_1–393_ was 0.8 nm larger than PTP1B_1–301_, suggesting that the C-terminal region is disordered and extended. NMR measurements further revealed that the C-terminal region was not completely disordered but contained two helical regions (helices α8′ and α9′). FRET measurements were performed by attaching the fluorophores to the N- and C-terminus of PTP1B_1–405_, and it was shown that in the presence of the inhibitor PTP1B attains a compact structure beyond a certain inhibitor concentration.

No NMR chemical shift perturbation was observed on addition of MSI-1436 to PTP1B_1–301_, suggesting the absence of a binding site in this region. In the case of PTP1B_1–405_, amino acid residues on the α9′ helix (see Figure 6) including other residues on the C-terminal disordered region showed perturbations. Helix-destabilizing mutations on α9′ had the largest effect on the binding affinity of MSI-1436, implying that the α9′ helix (Site1 in Figure 6) is the prominent binding site for the molecule. Mild inhibition of PTP1B_1–321_ by MSI-1436 was observed, suggesting the presence of an additional binding site in the catalytic domain (see Figure 6). An allosteric site was previously identified in the catalytic domain in a region between the α3, α6, and α7 helices (see Figure 6). Interestingly, mutation in the allosteric site led to a loss of inhibiting capacity of MSI-1436 for the full PTP1B. This suggests a role of the allosteric site in the mechanism of inhibition. However, the observed lack of inhibition of PTP1B_1–301_ suggested the presence of the additional binding site in the region of the catalytic domain between residues 301 and 321. NMR measurements showed that significant chemical shifts in the presence of MSI-1436 occur in the region of the α7 helix (Site2 in Figure 6) which implies that this helical region mediates the binding of MSI-1436 (see Figure 6). Notably, the α7 helix lies in the region between residues 301 and 321. Mutation studies indicated cooperativity in drug binding between Site1 and Site2. An interesting mechanism was proposed, where cooperative binding of MSI-1436 simultaneously to the α9′ and α7 helices (Site1 and Site2, respectively, in Figure 6) reconforms the allosteric site, where another MSI-1436 can then bind, albeit with low affinity (binding site is marked by a green circle in Figure 6). MSI-1436 binding to this site then allosterically inhibits PTP1B activity. The mechanism was supported by binding energy calculations from MD simulations and clearly explains the importance of the residues between 301 and 321 for inhibition. It also explains why the mutation at the allosteric site precludes inhibition of the full PTP1B.

### 4.3 Drugs Targeting Fuzzy Complexes: Precluding Nuclear Protein 1–MSL1 Binding

We now discuss the identification and development of a drug targeting a disordered protein involved in a fuzzy complex (a complex between two disordered proteins, not involving folding upon binding) with its disordered binding partner. Nuclear Protein 1 (NUPR1)^194^ is a disordered protein. One of the major functions of this protein is disruption of Kras^*G12D*^ (the gene producing the K-RAS protein that relays signals to a cellular nucleus with instructions on cell growth and division) function for modulating pancreatic lesions.^195^ The protein controls pancreatic cancer cell migration, invasion, and adhesion.^196,197^ It is overexpressed in pancreatic acinar cells in the acute phase of pancreatitis, and its depletion in cells featuring pancreatic ductal adenocarcinoma (PDAC) leads to cell-cycle arrest and senescence (cell death).^198^ These factors make it a possible drug target against PDAC. NUPR1 needs to interact with its disordered binding partner MSL1 in order to perform its function and is involved with MSL1 in a fuzzy complex. NUPR1 is mostly disordered, but some regions of its sequence attain transient folded structures. These transient structures may be stabilized by the protein interacting with potential ligands. Neira et al.^91^ performed high-throughput screening on FDA-approved drugs to identify molecules that interact with NUPR1. The identification of the compounds was carried out by studying the denaturation pattern of NUPR1 in the presence of 1120 FDA-approved drugs. Fifteen drugs were identified based on the difference between the thermal denaturation profiles exhibited in their presence, compared to the control sample in the absence of any drug molecule. Following this, isothermal titration calorimetry^199^ was used to measure the dissociation constants for the interaction between the 15 drug molecules and NUPR1 which were all found to be of the μM order. Then, an NMR-based investigation was performed to determine the binding region of the drugs on NUPR1. It was seen that the binding of the molecules to NUPR1 did not lead to any significant change in NMR chemical shifts of any cross-peak. This indicates that even in the presence of the drug molecules the protein mostly remained disordered, and the difference in thermal denaturation profiles observed during screening was a result of the interaction of the molecules to specific, localized regions in the NUPR1 sequence. They also analyzed the broadening of resonances of the 2D ^1^H–^15^N heteronuclear single quantum coherence (HSQC)^200^ spectra and found that there were small but consistent variations in the broadening of the signals for some specific residues resulting from an exchange of the drug molecules between the free and the protein-bound states. This observation suggests a specific binding of the molecules in regions around these residues. They further found that the *aa*s showing consistent (although small) NMR shifts in the presence of the ligands fall in regions that are found (by *in silico* analysis) to be the most hydrophobic and order-prone, implying that the binding occurs at specific hydrophobic sites on the NUPR1 sequence. It must be stressed that an estimate of binding specificity of the drug molecules would require additional MD-based studies, as discussed earlier for the cMyc–Max system. The binding to hydrophobic regions implies an entropically driven complexation where the shielding of the hydrophobic regions from water leads to a significant gain in entropy that can override any entropy loss due to structuring of the binding sites.

Finally, the complexes between NUPR1 and the 15 short-listed drug molecules were modeled using the following procedure: short molecular dynamics simulations were performed, starting from an extended NUPR1, and conformations from the MD trajectory with hydrodynamic radius close to those obtained from diffusion-ordered spectroscopy (DOSY) NMR measurements^201^ were extracted. These structures were used to perform molecular docking to generate models of the protein–ligand complex. Two of the drug molecules showed the highest binding energy of −6.5 kcal/mol. Interestingly the docked structures depicted these two molecules bound to the residue that showed the largest ^15^N chemical shift in the NMR experiments.

**Figure 7:**
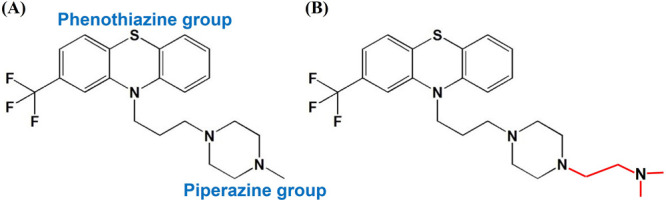
Chemical structures of (A) trifluoperazine and (B) ZZW-115.

Further tests show that the molecules hinder (to different extents) the interaction between NUPR1 and its binding partner MSL1 in a dose-dependent manner. The binding affinity of the molecules toward NUPR1 was comparable to that of MSL1. It was further found that one of the compounds, trifluoperazine (TFP)^202,203^ (see Figure 7(A)), inhibited PDAC cell growth, inducing cell death and decreasing chemoresistance similar to NUPR1 deficiency. The molecule however was found to cause neurological effects. To overcome this, recently, ligand-based drug design has been used to develop the compound ZZW-115 (see Figure 7(B)) and related compounds which are all based on the TFP scaffold.^204,205^ The approach involves performing docking of TFP on short NUPR1 segments, followed by relaxation of the complexes by MD simulations. The equilibrated complexes were used to identify chemical moieties of TFP that are important for interaction with NUPR1. The phenothiazine and trifluoromethyl group attached to it were found to be important based on energy calculations. As the increase in the length of the propyl chain between the phenothiazine and piperazine groups did not contribute toward improving the docking energetically, the methyl group attached to the piperazine ring was selected as the site of modification to derive new molecules.^204,205^

### 4.4 Drugs Targeting IDP Aggregation

#### Building IDP-Specific Drug Libraries

The inhibition of IDP aggregation underlies the drug development strategy for various neurodegenerative diseases. Joshi et al.^206^ used a fragment-based approach to generate small-molecule libraries of molecules with possible effectiveness against aggregation-related diseases like Alzheimer’s and Parkinson’s. Their approach included identifying recurring chemical fragments in reported inhibitors of aggregation of Aβ, tau protein, and α-synuclein. Ligand repositories were then screened for molecules containing these fragments. They observed trends in the chemical nature and structural features of the molecules. For instance, the molecules in the library of Aβ aggregation inhibitors contained a terminal phenyl group and aromatic end group with hydroxyl substitution. A subgroup of the molecules with representative chemical architectures, in the Aβ42 library, were experimentally tested for their effect on fibril formation. Out of 15 compounds, 3 resulted in a reduction in aggregation, throwing more light onto the possible chemical nature of potential inhibitors of aggregation.

#### Understanding Inhibition of Aβ Aggregation

**Figure 8:**
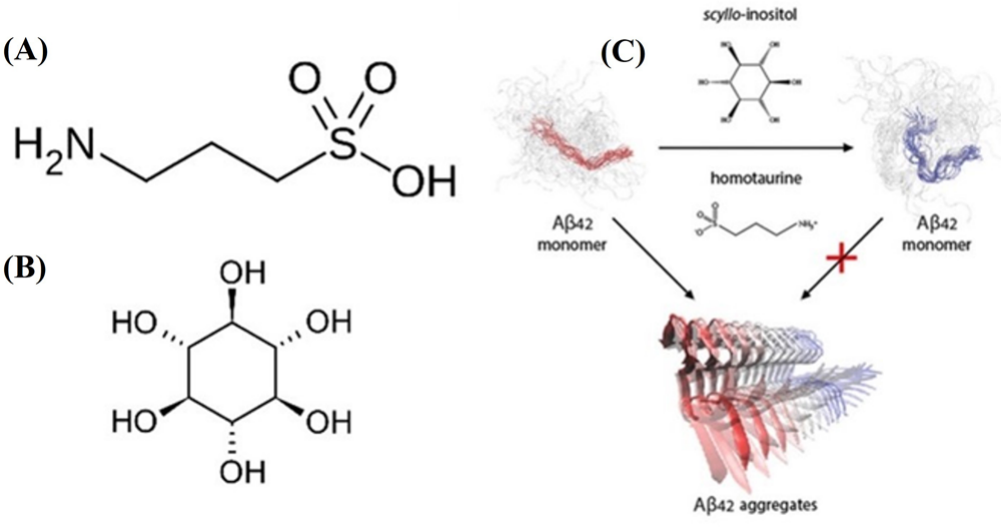
Chemical structures of (A) homotaurine and (B) scyllo-inositol. (C) Schematic depicting the main result from the work of Liang et al.^117^ Reprinted (adapted) with permission from *J. Chem. Theory Comput.***2019**, *15* (10), 5169–5174. Copyright 2019 American Chemical Society.

Liang et al.^117^ studied the mechanism of inhibition of Aβ aggregation by small-molecule homotaurine^207,208^ and scyllo-inositol^209,210^ (see Figure 8). They used MD simulations to analyze the interaction between monomeric Aβ peptide and the molecules and the resulting shift in the conformational ensemble of the peptide.

The CHARMM36m *ff* was used in combination with TIPs3P *wm*, while the small molecules were described with CHARMM Cgenff.^211,212^ Enhanced sampling was performed with the REST method described earlier. Sixteen replicas were simulated with temperatures in the range 300–640 K, and each replica was simulated for 10 μs. The convergence of the simulations was ascertained by comparison with NMR data. The C-terminal region was seen to attain a more collapsed conformation on average in the presence of the small molecules. While the major conformational forms stayed the same, the weight of the C-terminal conformation shifted from a kinked to a C-shaped form (see Figure 8). The C-terminal region has importance in Aβ aggregation. A shift in the equilibrium weights of different conformations would lead to a change in the tendency to aggregate. Inhibition of α-synuclein aggregation by cNDGA^213^ was also attributed to a shrinking of the conformational ensemble of the IDP by the small molecule, mediated by transient interactions.

### 4.5 Disrupting Disordered-Folded Complexes: The EWS-FLI1–RHA Complex

There are examples of IDP-folded protein interactions in disease propagation pathways where a disorder-to-order transformation upon binding is not well established. Ewing’s Sarcoma is a cancer originating in the bones and the tissues surrounding the bones, mostly affecting children and young people. The symptoms of this cancer range from pain in bones to broken bones even without an injury.^214^ The pathway to the development of Ewing’s Sarcoma involves translocation of chromosomes that encode mutated fusion transcription factors.^215,216^ The mutated fusion transcription factor associated with Ewing’s Sarcoma is the disordered protein EWS-FLI1^217,218^ which is formed as a result of the fusion of the N-terminal half of the RNA-binding protein EWSR1 and C-terminal half of the DNA binding domain of the ets-family transcription factor FLI1.^219^ The C-terminal domain (from FLI1) of the fusion protein is mostly ordered (except the extreme C-terminal end), and most of the disorder is restricted to the N-terminal domain (from EWSR1) which has been found to have very high intrinsic disorder propensity.^220^

EWS-FLI1 binds to DNA and interacts with the transcriptional machinery by binding to multiple protein partners. The protein binding partners of EWS-FLI1 include RNA polymerase holozyme II, RNA helicase A (RHA), and CBP among others.^221^ The binding of EWS-FLI1 to RHA is thought to be a critical step in the development of Ewing’s Sarcoma.^84,222^ RHA has an important role in embryogenesis and is a transcriptional coactivator in tumorigenesis models.^223^ The N-terminal region of RHA is the site for binding of other proteins forming essential functional complexes. Interestingly, EWS-FLI1 binds RHA along a region that is not occupied by other binding partners of RHA, rendering this protein–protein complex a possible target of appropriate small-molecule drugs. Erkizan et al.^84^ used surface plasmon resonance (SPR)^83^ to screen 3000 small molecules for binding to EWS-FLI1.

**Figure 9:**
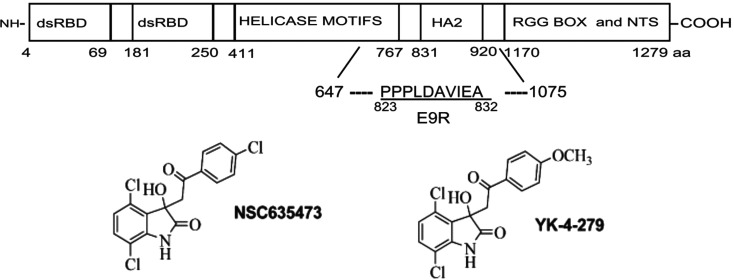
Schematic representation of the RNA helicase A (RHA) sequence with the position of the E9R peptide region indicated, along with the chemical structures of YK-4-279 and the parent compound NSC635473. Adapted with permission from *Nat. Med.***2009**, *15*, 750–756.

They closed in at the molecule NSC635437 (see Figure 9), based on SPR-derived binding affinity, as a potential drug candidate. The molecule was seen to reduce RHA–EWS-FLI1 binding. A derivative of the molecule, YK-4-279 (see Figure 9), obtained by substituting with a methoxy group at the para position of the aromatic ring of NSC635437 significantly reduced the interaction between the two proteins. YK-4-279 also exhibited functional inhibition of EWS-FLI1 in Ewing’s Sarcoma cells. It was also seen that the molecule can displace the peptide E9R (see Figure 9), derived from the HA2 region of RHA, from EWS-FLI1. The peptide E9R, on its part, is known to compete with RHA in binding to EWS-FLI1. This suggests a higher efficacy of the small-molecule drug as compared to the possible peptide drug E9R. Additional experimental studies on EWS-FLI1–RHA complexation could help understand whether the binding is associated with a disorder-to-order transition. Similarly, additional experimental and computational studies can help establish the mechanism of action of the drug, especially whether the drug binds to the folded or the disordered region of the fusion protein.

### 4.6 Structure-Based Drug Design

**Figure 10:**
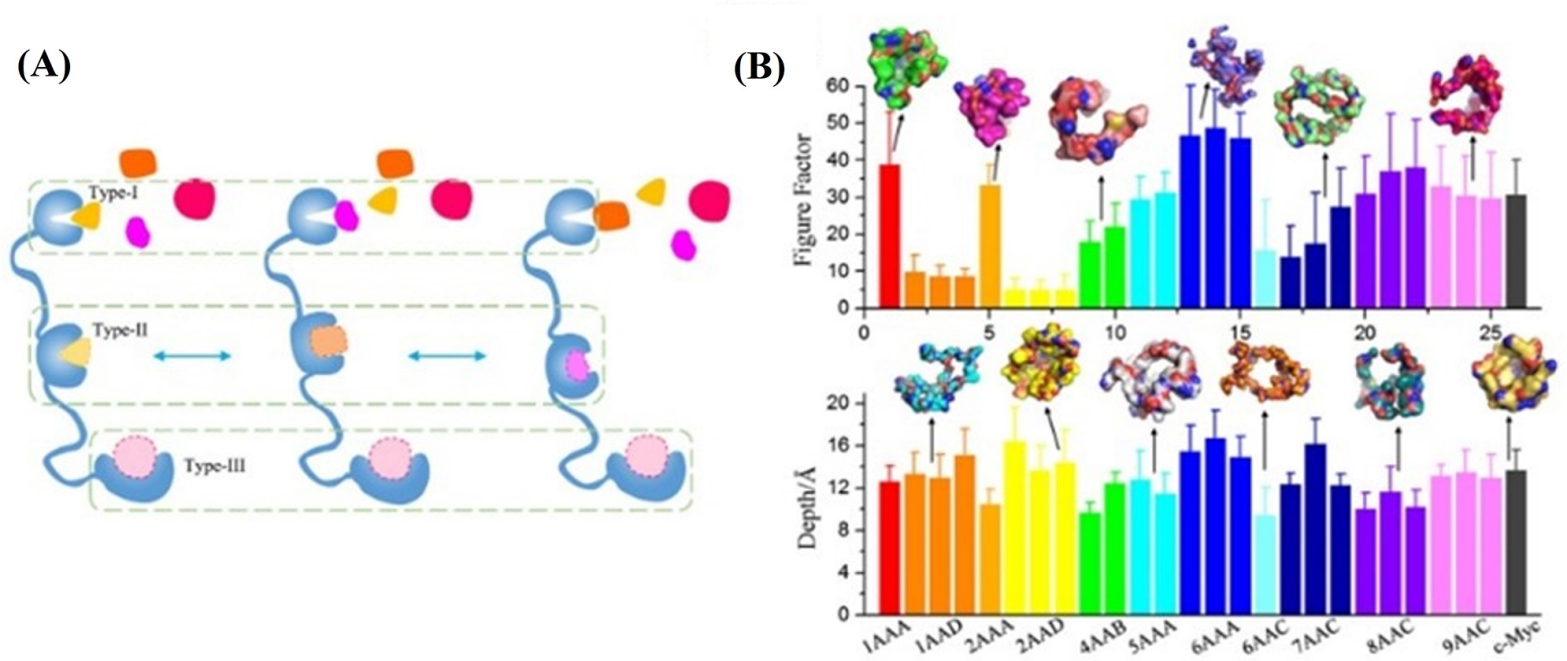
(A) Three types of disordered protein cavities. Type I corresponds to conserved but nondruggable cavities; Type II are the cavities that are not conserved but are druggable; while Type III are the cavities that are conserved and druggable.^224^ (B) The figure factor and maximum depth of cavities corresponding to structural ensembles of different proteins (ensemble names mentioned along the *x*-axis). The figure factor measures the deviation from a circle of the 2-D projection of the cavity shape on a plane perpendicular to the direction of maximum cavity depth.^224^ The *x*-axis is the pE-DB ensemble id. Different bars of the same color correspond to different clusters of cavities (see text) within an ensemble. Reprinted (adapted) with permission from *ACS Omega***2018**, *3* (11), 15643–15652. Copyright 2018 American Chemical Society.

Rational drug design involves identifying functionally important drug-binding pockets on the protein surface and identifying or designing small-molecule drugs that can bind to the pockets efficiently to block certain processes either directly or allosterically. However, as seen in the examples discussed in the preceding sections, for drugs targeting disordered proteins, the mechanism of action varies from binding to specific hydrophobic sites along the sequence to binding to multiple conformations and modifying the conformational ensemble through a “ligand cloud around protein cloud” description. Such a mechanism precludes a rational approach to drug design. However, modification of the conformational ensemble could be of two different kinds. Either the drug molecule can force the protein into acquiring entirely new conformations,^225^ or it can modify the conformational space by increasing the weight of some recurring conformations of the free protein ensemble.^226^ In the latter case, the drug preferentially binds to some suitable conformations of the free protein. Searching for such recurring conformations can provide transient binding pockets to work with and design drugs that fit into them and bind favorably. Chong et al.^224^ performed computational analysis of NMR and SAXS generated conformational ensembles of IDPs deposited in the ensemble repository pE-DB,^227,228^ using the CAVITY program,^229^ to identify druggable cavities and calculated properties related to their shape and size (see Figure 10). They found a small variance in the surface area and volume of the druggable cavities within an ensemble, while the difference in the average values was considerable between different ensembles. The cavities in the IDPs were found to have a larger surface area, volume, and cavity depth:  (*V*: volume; *S*: surface area), as compared to those in folded proteins, suggesting the possibility of strong ligand binding. Cavities were clustered based on their average position along the sequence (determined by taking an average of the positions of the atoms constituting the cavity along the sequence of the IDP), and parameters determining the degree of conservation were calculated for each cluster. The shape conservation of the cavities in each cluster was measured from the standard deviation of the cavity depth and figure factor (see Figure 10). The compositional conservation was determined by calculating the percentage of atoms common between the cavities in a cluster, which was found to be very high (>50% for many clusters), suggesting that the shift of potentially druggable cavities among conformations is small and that the druggable cavities are conserved among different conformations, thus presenting the possibility of a rational drug design approach. The cavities were found to have a high binding affinity toward test ligands.

Yu et al. used a variant of this approach to identify inhibitors of cMyc–Max binding. They performed MD simulation of cMyc (*ff*:AMBER99sb; *wm*:TIP4P-ew) to generate conformational ensembles. Representative conformations were selected out of the MD trajectory. Molecules were virtually screened by performing docking to these conformations. Selected molecules were seen to bind to cMyc directly, and some of them could inhibit cMyc–Max binding.

## 5 Discussion and Perspectives

The examples discussed above demonstrate that the disordered proteins and regions involved in disease propagation pathways can be targeted. Drug discovery targeting disordered proteins utilizes an approach different from that used for folded proteins. A combination of experimental methods are used in synergy to determine the binding sites and mechanism of action for small-molecule drugs that target disordered proteins and regions. MD simulations were seen to be crucial to unravel details of drug–IDP binding that are beyond experimental resolution. Unlike drug molecules that target folded proteins, those targeting IDPs demonstrate restraining or broadening of the IDP conformational ensemble as a mechanism of action in addition to blocking target binding sites on the IDP through specific binding to regions along the sequence. Thus, the structural plasticity and the sensitivity of IDP conformation toward environmental factors (presence of the drug molecule in this case) is utilized by drug molecules to affect IDP structure and interaction. The molecules targeting IDPs show affinity toward hydrophobic residues and regions along the sequence, resulting in entropically driven binding. Simultaneous binding to a combination of such regions allows the drug molecules to affect the IDP conformational ensemble. The differences in approach to drug discovery targeting folded and disordered proteins originate due to the absence of stable and well-defined binding pockets in IDPs and IDRs. Rational drug design for folded proteins involves techniques like molecular docking and *de novo* design, which are applicable because a clear picture of the target structure is available from experiments. Determining the conformational features of IDPs/IDRs requires a greater synergy between experimental and computational methods because, unlike in the case of folded proteins, experimental techniques alone cannot provide uniquely interpretable results regarding the conformational ensemble of IDPs. Moving toward a rational approach to drug design would involve determination of sequence characteristics of regions that drug molecules (of a broad range of chemical nature) show affinity toward and determination of quantitative structural parameters. Determination of amino acid pairs along the IDP sequence, for instance, which undergo correlated motions, could facilitate the design of molecules with specific chemical and spatial parameters which could interact favorably with the IDP. Identifying druggable cavities that are conserved over mutiple IDP conformations, as discussed before, is another plausible approach toward setting up a rational drug design protocol for IDPs. Identification of recurring structural features would inspire a wider use of techniques like docking and *de novo* design in the context of disordered drug targets. Such approaches, in addition to accurate experimental techniques, would require robust computational methods. In fact, MD simulation derived docking scoring functions have recently been defined that can successfully reproduce experimental ligand–IDP binding preferences,^230^ enabling structure-based drug design for IDPs in addition to the ligand-based approaches in use.^204,205^ An MD simulation based virtual screening protocol has been developed where a structural ensemble of the IDP is generated by simulations. Conformational clustering is then used to generate a set of binding pockets/cavities into which the ligand is then docked. Ligands that can bind multiple binding pockets are then selected for further tests. The approach has been used to develop compounds that can bind the TAD1 domain of p53.^231^ While the computers in their current state are fast enough to perform long simulations of IDPs in combination with enhanced sampling methods, a further improvement in the quality of the IDP-specific *ff*s and also of *ff*s that can describe both folded and disordered proteins simultaneously is desirable. In addition to the recent developments in force fields mentioned in this article, coarse-grained force fields like MARTINI3 have been modified to simulate disordered proteins, using a strategy similar to that used for the development of the TIP4P-D water model, namely, modifying the protein–water interaction to get rid of overcollapsed IDP conformations.^232^ While such developments are interesting, the coarse-grained force fields have a long way to go before they can appropriately reproduce the transient structural properties of disordered proteins. The recently developed hybrid resolution (HyRes) protein model^233^ and subsequent modifications to it^234^ are important steps in this direction. Ligand-structure analysis based protein-specific small-molecule libraries, similar to that attempted by Joshi et al.,^206^ would be helpful to carry forward drug discovery through a rational approach. In combination with experimental techniques, computational methods can help with an unambiguous understanding of the working mechanism of various drug molecules, which would pave the way toward improving the performance of drugs through rational modifications.
